# The coproduction work of healthcare professionals in police custody: destabilising the care-custody paradox

**DOI:** 10.1080/10439463.2022.2055020

**Published:** 2022-03-28

**Authors:** Gethin Rees

**Affiliations:** School of Geography, Politics and Sociology, Newcastle University, Newcastle Upon Tyne, UK

**Keywords:** Healthcare professionals, police custody, deaths in custody, coproduction

## Abstract

Forensic medicine has traditionally been understood as constituting a tension between medical and legal roles: a care-custody paradox. Rather than reinforcing this paradox, however, in this paper I will draw upon a study of Healthcare Professionals working within police custody suites in England in order to show the ways that they coproduce [Jasanoff, S., 2004. *States of knowledge: the co-production of science and social order*. London: Routledge] their work with the aim of simultaneously meeting the requirements of both their police (for instance PACE codes) and healthcare (for instance the Nursing and Midwifery Code of Practice) responsibilities. Focusing on acts of ‘mundane care’ [Brownlie, J. and Spandler, H., 2018. Materialities of mundane care and the art of holding one’s own. *Sociology of health and illness*, 40 (2), 256–269], the typification of detainees and the use of detention cells as risk management tools, I will show that rather than undergoing an existential crisis, Healthcare Professionals mobilise coproduced practices in order to perform their work successfully, thereby further enabling police and detention officers to achieve their custody objectives.

## Introduction

In this paper I aim to reconceptualise the ways that healthcare is understood within police custody suites. Traditionally, scholarship around forensic medicine has emphasised its contradictory nature, postulating conflicts either on the part of the lone forensic professional attempting to reconcile their healthcare ethics with working as part of a criminal investigation (Kelly *et al.* 1996, 1998, Savage *et al.* 1997) or at a professional level, where the attitudes and approaches of embedded Healthcare Professionals^1^ (HCPs) are considered at odds with those of their police colleagues (De Viggiani 2013). It is my contention that this care-custody paradox has been overstated and that HCPs instead coproduce (Jasanoff 2004) their work in order to meet the requirements of both their police (for instance Police and Criminal Evidence – hereafter PACE^2^) and healthcare responsibilities (for instance the Nursing and Midwifery Council professional codes of practice). In so doing, they help their police and detention officer colleagues accomplish their broader custody objectives.

Reconceptualising forensic healthcare is timely, as scholarship has begun to reflect on the purposes of police custody (Skinns *et al.* 2016). The police custody space acts as a gateway to the criminal justice process, and is significant for the collection of evidence, but can also be seen as: a place for reforming an offender; a space of safety; and a place where staff safeguard future legal processes by managing detainee-based risks (for instance diagnosing and treating relevant healthcare concerns). The work of Skinns *et al.* (2016) highlights the multiplicity of objectives negotiated by the police custody team (both legal and welfare) and, importantly, draws attention to the role that HCPs play in ensuring the custody suite team successfully achieves these ends.

Against this background of situating healthcare delivery within the complex and multipurpose space of police custody, I want to challenge the assumption that forensic healthcare exists within a care-custody paradox. Separating healthcare responsibilities from custody activities fails to identify the ways in which such practices are necessary to accomplish criminal justice tasks. Moreover, it can also serve to idealise the therapeutic aspects of care work, failing to acknowledge the potential harms that can result. In this paper, drawing upon work from the Sociology of Health and Illness, especially recent work on the materialities of care (Buse *et al.* 2018), I will explain how care work can be strategic, mundane and punishing, as well as supportive and therapeutic. As such, rather than healthcare in police custody being juxtaposed to police work, coproduced healthcare should be seen as integral to the successful accomplishment of police custody work.

## Literature review

### The care-custody paradox

Traditionally, scholarship around forensic medical work has emphasised a clear distinction between evidence-gathering and care responsibilities, with practitioners described as experiencing a constant state of role-conflict between their everyday therapeutic considerations and the requirements of evidence generation for the police (Savage *et al.* 1997). One practice utilised to exemplify this role-conflict was the treatment of women reporting sexual assault (Kelly *et al.* 1998). In performing post-sexual assault forensic interventions, police doctors claimed to be uncertain as to who constituted their ‘client’: the police or the victim-survivor. As the victim-survivor was not the doctor’s patient (in terms of their everyday General Practitioner role), they did not consider themselves to have an ongoing duty of care, and therefore emphasised evidence-gathering (for instance, taking invasive and harmful samples) due to their ongoing relationship with police staff.^3^ The tension between care and evidence-generation was resolved by adhering to the police role.

More recently, De Viggiani (2013) has expressed a similar argument, albeit framed within the ethical dilemmas faced in police custody following the introduction of commissioned nurse-led programmes. He suggests that the differing professional values between healthcare and the police necessitate that HCPs advocate for detainees,^4^ but this might in turn prove problematic for professional relations within custody. He proposes a public health model where all detainees are risk-assessed by HCPs in order to avoid potential tensions with police (De Viggiani 2013). While De Viggiani’s work is different to that of Savage and others in the sense that the practitioner does not experience a personal role-conflict, it still assumes a clear distinction between police and healthcare around the purpose of police custody, something the evidence of Skinns *et al.* (2016) does not support. There is therefore a clear tradition within the (predominantly criminological) literature that argues for a distinction between care and custody work. They are considered mutually exclusive categories, and HCPs are positioned solely as care providers.

The dialectical relationship between care and custody has been challenged in humanities and social scientific scholarship. Focusing on professional practices, the role-conflicts expressed by forensic professionals disappear when we observe or discuss routine work in detail. For instance, Sexual Assault Nurse Examiners follow guidelines when deciding whether to collect forensic samples; in this way, they generate good quality evidence while also minimising further harm (Mulla 2011, Rees 2015a). In this analysis, a role-conflict does not so much constitute a decision-point within everyday work but rather a rhetorical tool applied, for instance, as a response to critical attacks from the fields of either medicine or law (Bourke 2018). Similarly, role-conflict discourse is useful when asked about work by an interested other, for example a Judge during expert testimony (being able to situate one’s practice as wholly medical can serve as a way of generating credibility (Rees 2010)), or when interviewed by a social scientist.

This is not to say that important ethical concerns are simply rhetorical constructions; issues such as deciding which types of samples to take in post-sexual assault interventions or ascertaining how to maintain the dignity of a sick person in custody certainly require complex decision-making. The point is that forensic professionals have found ways to mix together legal and therapeutic responsibilities, coproducing (Jasanoff 2004) new routines, guidelines and practices that, while neither wholly criminal justice or medical, enable both sets of standards to be achieved simultaneously.^5^

### Dignity and the purpose of custody

A similar discussion about the balance between care and custody has recently been instigated by the ‘Good Custody’ project by Skinns and colleagues (see for instance Skinns *et al.* 2017, Wooff and Skinns 2018, Skinns and Wooff 2020). In a recent article, Skinns *et al.* (2020) attempted to define ‘dignity’ and ascertain how dignified practices could be operationalised within police custody. A key aspect to dignity, they argued, involves the treating of detainees as human beings. There are two reasons why police and detention staff choose to treat detainees with respect: first, there is an acknowledgment that detainees are deserving of respectful treatment; and secondly, pragmatically, getting detainees ‘on side’ is a way to enable compliance and thereby generate a quieter shift in order to get on with work (Skinns *et al.* 2017). Skinns *et al.* (2016) noted that the custody environment is: a space for reforming young offenders; a place of safety; a place to collect evidence; and a place to safeguard the legal process by successfully risk-assessing, diagnosing and treating healthcare requirements, as well as maintaining compliance with other PACE responsibilities. Given the sometimes-conflicting nature of these tasks (for instance the tension between the PACE clock and ensuring a person has a place of safety upon release), making custody a quieter space helps with the accomplishment of these goals. These discussions about dignity, respect and the purposes of custody clearly highlight the importance of sensitive care work by the custody team, but also, importantly, emphasise the role of care, and especially healthcare, in achieving these various custody objectives. To explain care, I will draw upon recent literature from the Sociology of Health and Illness.

Care activities extend from highly technical life-saving practices to small-scale acts of interpersonal compassion and intimacy. Brownlie and Spandler (2018) recently studied these latter acts, investigating the ways that certain activities (e.g. fetching someone’s shopping) can constitute mundane acts of care, allowing the recipient to feel identified and their needs recognised (Brownlie and Spandler 2018). The authors understood mundane care as the products of networks of actors embedded within, in their case, a ‘moral economy shaped by familial, local and societal expectation’ (Brownlie and Spandler 2018, p. 256). In the broader community, acts of mundane care (for instance walking an elderly neighbour’s dog) might be based upon interpersonal relationships or the belief that elderly persons should be treated with respect. However, in police custody, where a pre-existing relationship between the detainee and HCP might not exist, acts of mundane care require other explanations, for instance pragmatism or risk-management. Similarly, work on the dressing of persons with dementia (Buse and Twigg 2018) has highlighted the high quantity of emotional work necessary in that context, which is itself an act of mundane care. The carer needs to present the correct emotional attitude in order to develop rapport, and to empathise with the needs of a person who is required to dress in front of them. These studies are certainly resonant with discussions in custody, where questions of dignity have previously been framed around the use of rip-proof paper suits and the nudity of detainees (Skinns *et al.* 2020), as well as rapport generation (Wooff and Skinns 2018).

Care is a practical activity, but is also mediated by artefacts and spaces (Buse *et al.* 2018). The importance of this point was exemplified in Bell’s (2018) study of migrants accessing hospital healthcare. The architecture of waiting rooms can enable sociality by providing spaces for conversations between attendees, but this also risks confidentiality, especially if medical staff request information or additional samples while in the waiting room. Conversely, the design of waiting rooms can also produce feelings of anonymity and isolation. Wooff and Skinns (2018) have made a similar point, emphasising the ways that the design of police custody environments produce a sense of isolation for the detainee, as well as liminality (the absence of windows and clocks, for instance, remove a detainee’s sense of time). Material resources, for instance food, clothes, bodies and the design of spaces, therefore have important affordances and limitations in the delivery of care.

### Care as typification work

Thus far, care has been presented in opposition to the harsher or more controlling aspects of custody, and as a means to provide kind and dignified treatment. In this section, I will take a more cynical approach, highlighting the ways that care produces typologies of patients (Latimer 1997). Healthcare professionals classify patients on first interaction via triage mechanisms, prioritising certain kinds of patients over others (Johannessen 2018). This classification shapes, and is shaped by, the organisational context and policies within which persons are given care (Latimer 1997). Situated between potentially contradictory policy objectives, the way a patient is classified and their care trajectory could alter depending on the priorities of the institution. For instance, a patient’s dignity and autonomy might be limited by a forthcoming surgery, treatment regime or research project within which they have been included. Like the hospital environment, police custody can be located amidst a range of oftentimes conflicting objectives, and the operationalisation of these will impact on the classification and care of the detainee.

Healthcare is a necessary aspect of custody work, and its importance is often overlooked. Below, I will highlight police custody healthcare activities, showing how HCPs coproduce their work to balance legal and medical responsibilities. In doing so, I intend to promote a new conceptualisation of police custody healthcare: one that destabilises the care-custody paradox.

## Methods

This paper derives from an exploratory project investigating the challenges faced by nurses when working within police custody. Semi-structured interviewing was chosen as the data collection method; while it was appreciated that observation provides a better method for understanding working practices (Allen 1997), it was considered impractical given the exploratory nature of the study and the short period within which it needed to be completed. As a result, nurses working within custody were recruited and interviewed.^6^ Access was generated via the United Kingdom Association of Forensic Nurses, who advertised the research project to their members. 24 nurses responded to the advertisement and 20 were interviewed, with the other four withdrawing, often due to difficulties with finding a mutually suitable time. As a result, the respondents represented seven constabularies in England (out of 39), and the interviews took place between 2017 and 2018. Four of the constabularies employed private providers, and so the nurses were employees of private companies (and the four constabularies represented three different providers). Two constabularies had a contractual relationship with the NHS to provide healthcare, and in one constabulary the police directly employed HCPs. Of the 20 interviewees, 13 were employees of private companies, four were NHS employees and three were police employees. Three of the respondents were men, and length of service as a custody nurse ranged from 19 months to 13 years (mean eight years). The interview schedule was structured around the following themes: the respondents’ background, training and entry into the HCP role; a description of an ordinary day in police custody; governance of HCPs (payment, conditions and covering shifts); and preparing legal cases (for instance statement writing, giving testimony in court). All interviews lasted between one and two hours and were digitally recorded and transcribed verbatim. The project was approved by Newcastle University’s Humanities and Social Science Research Ethics committee.^7^

Once collected, the data were analysed solely by the author using Framework Analysis (Ritchie and Lewis 2004), with the inductive development of core and subsidiary themes. The transcripts were indexed and the interview sections placed into a Framework Matrix in order to assess the similarities and differences between respondents, especially those working under different governance procedures (e.g. private or public providers). Themes were continually evaluated for their utility during the indexing process, with Framework Analysis constantly enabling the contextualising of themes and ideas within the original transcription, something that is not always possible with other qualitative analysis methods (Ritchie and Lewis 2004). Key themes included mundane care, the classification of detainees, and the spatiality of the cell.

## Analysis

### Mundane care

The role of nurses working within police custody is to ensure the health and wellbeing of those detained. While the custody sergeant ultimately has responsibility for all those in the custody suite (detainees and staff), HCPs provide medical expertise in order to assist the sergeant in making decisions about a person’s fitness to be detained or interviewed and to provide assessments of medical needs during the period of detention (Rees 2020). Key to HCP decision-making is the clinical assessment of a detainee, as this will form the basis of any further healthcare interventions during the period of detention. One interviewee, Naomi, described how she introduced the assessment in order to explain her method of rapport generation.

I don’t have any judgments about these people, apart from the fact that they are there and my job is to look after them … I always treat people with respect and I always say to them “Is it okay for me to call you” whatever their first name is, “My name is Naomi” and so if you do things like that it breaks down barriers right from the start. (Naomi F NHS)^8^

Similarly, Harriet pointed out:

Yeah, you are appearing caring and showing interest in them as a person rather than just … tick, tick, tick, I give you that, which is what I can clearly show to them, and there is more to people than just that. (Harriet D2)

Taking an interest in the detainee and talking to them courteously is not only a professional practice but also allows the interlocutor to feel respected, which is appreciated (Skinns *et al.* 2017). As an example of mundane care, holding a conversation with a person in a personable and polite manner generates rapport with and engagement from the detainee. However, displaying a caring attitude here is not simply altruistic; it is also strategic.

As well as personability as rapport-building, nurses commented on the potential for acts of aggression in custody: ‘a lot of people who are arrested in custody, the lack of control is a huge issue for them, that’s why they kick off, cause problems, because they’re trying to get that control back’ (Eleanor D2). Similarly:

We have a different client group from what you’d get in hospital very often, you come across them in A&E, but you get a mixed range: in A&E you get the crying child, you’ve got the sick mum and you’ve got the ill teenager, drunken but they are not necessarily expecting to be, in the hospital, they are more likely to be quiet because of the institutional setting. Um, when we get people, they are already antagonistic because they’ve been brought in by the police, because they come with expectations and challenges, usually high on drugs or alcohol. (Harriet D2)

The healthcare assessment is therefore understood as a difficult and potentially risky activity, and politeness is a means by which to accomplish the task without potential harm.

[B]ut I think it’s just nice for patients to know that, because they don’t know what time it is, they’re pressing the buzzer, nobody’s going to them, then they come to me and they’re going to vent for ten minutes at me and it’s not my fault, so I try and keep the patients happy. (Catherine B2)

A detainee’s liminal status (Wooff and Skinns 2018) and lack of control produces risks that can be managed by treating detainees with dignity and respect; in so doing, the HCP is able to complete the healthcare assessment.

Other mundane expressions of care (Brownlie and Spandler 2018) were evident in the work of the nurses, for instance the decision to collect a detainee’s medication on their behalf while they were in custody.

I also don’t like the idea of setting someone up to fail, so for example, if someone has come in on a weekend, on a Saturday or been arrested Friday night, but they look like they are going to be released Saturday at some point but it’s past six o’clock when the chemist is shut, I will try and liaise with the pharmacy, because they have to pick up a whole weekend’s script “Due to pick up for the weekend, won’t be out in time, would you be able to hand over to a police officer or myself if I come in and collect it?” so at least when they release from us they have that dose for the day. (Lucy E Police)

Lucy presented the collection of a detainee’s prescription, often relating to methadone prescribed as part of a recovery programme, as an altruistic practice, ensuring that they have their medication for the next day and are not further disadvantaged by their period in custody. When presented in this way, collecting a detainee’s prescription, like collecting a neighbour’s shopping, is wholly consistent with a mundane act of care (Brownlie and Spandler 2018), enabling a detainee to feel ‘looked out for’ (Buse *et al.* 2018, p. 248). The same practice also serves another purpose within custody, however, as the police are responsible for the welfare of detainees for a period of time after detention. The collection of prescriptions was one means by which nurses mitigated future risk.

[I]t might be, we even collect their methadone or not … it is in the patient’s best welfare if they need that medication because they have run out and I need to make sure that their welfare is not just when they are in custody, but when they leave custody, so if, because of them being in here means that they have not had the medication, for safety then, with negotiation with the custody staff, we work together to try and get that for them to make sure that they are safe after they leave as well. (Harriet D2)

As Brownlie and Spandler (2018) have emphasised, mundane acts of care are the products of networks of expectations. Both examples of mundane care provided here – polite interactions with detainees and collecting prescriptions – can be understood as simple acts of looking out for a detainee’s wellbeing. At the same time, however, they also serve more pragmatic goals, such as generating compliance to aid completion of the clinical assessment, or reducing risk to the detainee upon release.^9^ The expectations here are therapeutic care and managing the risks potentially embodied by the detainee. In this way, these mundane acts do not constitute care or custody practices, but are instead coproductions that meet both ends simultaneously.

### Care as typification work

A clear distinction was made by the HCPs between alcoholics and drug-dependent detainees. From the beginning of their training, it was emphasised that there was a relatively high risk of death from alcohol withdrawal compared to the low risk from drug withdrawal.

Yup, in your training one of the first things you are taught is that heroin users do tell lies, and not so much our alcoholics, they are the ones that we look after more carefully perhaps, because that is so very dangerous withdrawing from alcohol. Withdrawing from heroin, I’m sure is deeply uncomfortable, I don’t know, but not so mortally or morbidly dangerous as withdrawing from alcohol, they are the ones we wonder about. (Fiona D2)

What we are trying to avoid is a death in custody, can’t be killing somebody by withdrawing pain relief, withdrawal medication for alcohol relief is different. That’s one of our mottos, “You don’t die of drug withdrawal, but you can die of alcohol withdrawal”, and that is a quote I quite often use with people. (Imogen D2)

The relative risk of death in custody was the central pillar for a series of other characteristics that HCPs used to construct the two different kinds of detainees with substance dependency issues. Alcoholics were constructed as quiet, low-maintenance and vulnerable:

People who are withdrawing from alcohol die and they die quickly, if you don’t see it, intervene and medicate them or ship them out to A&E and historically, the figures show that that has been a lot of the incidences of death in custody have been around not identifying withdrawal … Someone withdrawing and is alcohol dependent will quietly withdraw in a cell and you’ll suddenly go down and you’ll think “Oh my god, they are really poorly, they’ve got to go or they’re going to have a seizure and potentially die”. (Amanda A1)

Given the potential risk of a death in custody from alcohol withdrawal, nurses were keen to ensure that detainees were regularly provided with medication so that they would not enter a state of withdrawal, and also liaised with police staff to ensure that even if the detainee were not sober, they would be sufficiently functional to be interviewed and then released so that they could secure further alcohol. Amanda continued:

… and it’s then being very clear with the officers that are going to be interviewing, you have two hours to get whatever you need because then they’ll be coming off fit to detain, because I won’t be keeping [them in] if they are withdrawing massively from alcohol … we’ve got a couple of regulars and we know that if they are going to withdraw, they are going to withdraw big, so you’ve got four hours, clock’s ticking, come on boys.^10^ (Amanda A1)

Denise similarly mentioned that she had developed an expertise in knowing when an alcoholic detainee was sufficiently functional to be interviewed:

You’ve got to take into consideration their physical wellbeing, if it’s an alcoholic is it that this period of time, I’m desperately thinking of a phrase to coin this situation, where they are not withdrawing but they are not intoxicated either, it’s a really strange window, it’s like a Twilight Zone really, between being bladdered and being ill, where you have to fit the process into. (Denise C3)

Concerns around the risk of an alcohol-dependent detainee withdrawing meant that HCPs constructed them as highly vulnerable, but at the same time were aware of the police’s need to gather evidence. To this end, nurses developed their own expertise around the space of safety, where alcoholic detainees were functionally capable of being interviewed but not wholly sober. In so doing, nurses coproduced categories (like Denise’s ‘Twilight Zone’) and practices (collaborating with investigators) in ways that enabled evidence to be collected whilst still acknowledging the health risks.

Those with drug dependencies, by contrast, were depicted as much less vulnerable. HCPs framed drug users as manipulative, drug-seeking and high-maintenance.^11^

With alcohol I am glad there is that fear, because your alcoholic is going to come into custody, lie under the blanket all night and get progressively worse; with [an] opiate user, the person will come in with a needle still in their arm and say they are withdrawing … They obviously have drug seeking behaviours so whatever you offer them they’ll want more. I had someone who had a respiratory arrest because of an opiate overdose, got given Naloxone, taken to hospital, came back and said “I need to see a nurse, I’m withdrawing,” and you think “WHAT!” (Martin D2, emphasis in original)

Due to the assumption that those with drug dependencies are manipulative and drug-seeking, nurses were highly reluctant to provide medication in the early hours of detention (in contrast to an alcoholic’s detention, where treatments were supplied in order to hold off withdrawal), as there was uncertainty about what the detainee had previously consumed and therefore a risk of overdose.

Also, it’s a case of if they have had something just prior to custody and I see them and I give them something for their withdrawal, in theory I’m overdosing them and it’s my registration, and I’ve been nursing a long time and I’m not prepared to lose that for anybody, so that’s why I am going to challenge them. (Gillian D2)

The construction of the manipulative, high-maintenance drug-dependent detainee relates directly to the professional status of HCPs. In cases of alcohol dependence, deaths in custody are the product of an omission of care, i.e. failing to spot that someone is withdrawing and hence failing to provide appropriate care. The death of a drug user in custody is more likely to be a result of providing inappropriate care, i.e. the HCP has over-medicated the detainee, producing an overdose. This not only impacts the custody suite as a whole, as it will instigate an Independent Office for Police Conduct investigation, but could also potentially result in the nurse losing their registration and their livelihood. In order to protect themselves, the detainee and the custody suite, nurses either challenge detainees on their recent drug use, as described by Gillian, or straightforwardly deny medication for the first six hours of detention (this was the policy in all but one of the constabularies I visited). The HCPs explained that denying medication was the clearest distinction between their custody practice and their previous healthcare work.

So there does seem to be an element of that, if you were a good nurse you’d look after me, why do you make me get uncomfortable, why do I have to be seen to be withdrawing before you will give me anything? I don’t really know, because I have to be able to prove to myself and the world that you were withdrawing, and you’re quite right, why do you have to score on my Clinical Opiate Withdrawal sheet, because I must be able to stand in court and say the reason I gave those dehydrocodeine was because he was withdrawing. (Fiona D2)

[A]nd I’ve found I’ve got better at that over the years, if someone says to me “I’m withdrawing from heroin,” you can just look at them and say “No, you are not,” which they hate obviously, but it’s about having that awareness and the confidence to say “No, you are not”. (Eleanor D2)

As Eleanor implied, saying no to a detainee was a skill that HCPs had learnt over time. Such activities connoted a different form of care from the kinds they had previously employed in other functions, for instance working in hospitals. They stated that they had to deploy a different skill-set in order to perform their custody tasks.

So, I think you do, you can become quite cold in this job in some ways, compared to other nurses in sort of “on the wards”, cancer, always seen as more empathetic because they are dealing with death and dying and some people have been there, so not saying we are not the same people, we are the same people, but put us in a different environment, our different skills will come out. (Harriet D2)

In contrast to the empathic representation of the ward nurse, an HCP needs to develop resilience and the ability to deny treatment until there is clear objective evidence of pain; this means that HCPs become distanced, or ‘colder’ than nurses working on the wards. Some nurses attributed their coldness to seeing ‘the worst of people all the time, not only because of the things that have brought them in there, their behaviour’ (Naomi F NHS), but also because their task was not to heal patients, but rather, in the coproduction of meeting healthcare and legal objectives, to ensure that detainees were fit enough to be detained and interviewed (Rees 2020). The framing of care practices (and the classification of detainees) performed by HCPs as part of custody work produced the distanced attitude described by Harriet.

### Care as spatial

HCPs also discussed the importance of the cell as a space for healthcare interventions. Similarly to Wooff and Skinns (2018), nurses were aware of the liminality and uncertainty caused by being locked in a detention cell and the ways that this could inhibit interactions and the completion of the clinical assessment.

[A]nd we’ll try and speak to the person with the door open, if we can, because sometimes it just takes a little bit of human interaction to bring them around. If you keep a caged animal, they are going to be pacing up and down, they are going to be acting out, so sometimes you’ve just got to say “Come on fella what’s happening? How are you feeling?” and sometimes you get the reaction you want, “So you’re going to behave and come to the medical room?”, “Yes” and we manage to get them to behave and have some semblance of assessment. (Catherine B2)

The nurses had many different opinions about where the clinical assessment should take place; some preferred to conduct the assessment in the cell for their own safety, but this was generally considered poor practice. Most nurses expressed the opinion that the examination should only take place in designated medical rooms, to protect confidentiality.

A lot of our nurses are quite passionate about, obviously seeing them in a cell regarding confidentiality and whatnot, but it’s no less confidential if they spill all their guts up, confess all their stuff in front of the Custody Sergeant, which then goes on camera … Apparently, we should see everyone in the medical room, unless we risk-assess. Now me and some of my colleagues are very professional, if they have warning markers, which means they are in for common assault, they’re drug-seeking, they’re volatile and violent, I am not going to take them into an environment where it isn’t safe … if they’ve got warning markers, and they’re in for a certain offence, I will nine times out of ten see them in the cell. (Jess E Police)

We rarely see people in cell, rarely, only if they are unwell and literally cannot walk to the medical room, so we rarely see people in the cell. Unless they are being really aggressive, in which case we will speak to them through the hatch, if we don’t think there is much wrong with them, or much we can offer, but most of the time see them in medical room, on our own, with door shut. (Owen G NHS)

Nurses were required to balance their own personal risk of being alone with a detainee in a medical room against the confidentiality of the detainee’s healthcare details. Owen presented a more positive outlook regarding use of the medical room, but he also concluded that a risk assessment would be required before a detainee was allowed out of the cell. One explanation for Owen’s different take is the fact that he was employed by the NHS. In NHS-provided constabularies, the police and NHS have developed clear guidance on the way embedded nurse work should be performed, notably requiring HCPs to remain within the medical room with the door closed in order to maintain clear boundaries between medical and police colleagues. Unlike under other governance regimes where HCPs have argued for the benefit of overhearing the checking-in process of detainees, as it helps to anticipate the needs of the police (Rees 2020), in Constabulary G the medical room was constructed as a space separate from the rest of custody. It makes sense, therefore, that under the NHS/Police guidance, aimed at emphasising the independence of the HCP, the spatiality of the medical room would be seen as the appropriate place for all healthcare interventions, with the cell being a police space except when dealing with the most unwell detainees. Nevertheless, within the space of the medical room, HCPs working under NHS governance continued to use the same coproduced practices I have discussed above.

One benefit of the healthcare assessment taking place in the medical room was that the transition from the cell to the medical room itself became part of the healthcare assessment. Nurses were able to view the detainee walking to the medical room and attain an overall sense of their wellbeing.

And now we’ve got a medical room, we used to have to see them in the cells because we didn’t have a medical room, so that makes them come out and that’s all part of the assessment, we can see them walk, see them talk, see their demeanour in the medical room. (Imogen D2)

This was seen to be particularly useful if a detainee was complaining of drug withdrawal, as watching them enter the room gave a good indication of whether the person was likely to require medication or not. Imogen continued:

I’m not saying I’m blasé about it, but you can, “I’m withdrawing from drugs”, “Really”, “Yes” and they are not shaking, they are not bending over in pain, when you are talking with them … I’m not going to make them wait, if I feel a two-hour wait would be detrimental to them then I would[n’t] do it, but if you are stoned out of your head there is no way I am going to give you extra painkillers. Based on your demeanour or my observations, I’ll make a clinical judgment. (Imogen D2)

If a nurse made the decision that a detainee would not be provided with medication, for instance if there was still uncertainty over whether the person had recently consumed opioids, the differential spaces of the custody suite were again used to manage the risks of informing the detainee that they would not be receiving medication. Given the potential for the detainee to respond angrily if their requests for medication were denied, nurses waited until the detainee was back in the cell before informing them.

[A]nd I never say no to people in the medical room either, if I am going to say no about a medical decision I’ve already made in the first five seconds of them sitting down I always wait until they’re back in the cell before I say no to them. (Catherine B2)

HCPs therefore used the different spaces of the custody suite to manage the perceived risks to themselves posed by the detainees. Any detainee who was considered a risk was more than likely to be clinically assessed in the cells; although this breached the confidentiality of the disclosure of healthcare information, it was considered acceptable, as it was akin to what was disclosed during the desk risk assessment. When detainees were brought to the medical room, the transition was included as part of the clinical assessment. This enabled the HCP to assess the demeanour of the detainee, especially if they were claiming drug withdrawal, as that could be challenged based on the nurse’s observations of them walking and sitting. If the nurse decided she would not medicate, the detainee would be informed when they were back in the cell, as this reduced the risk of potential violence and harm. Similarly to Bell’s (2018) findings regarding hospital waiting rooms, it can be seen that spatiality of care provides limitations and affordances for both detainee and HCP; while the opportunity to leave the cell might break the monotony of detention for detainees (Wooff and Skinns 2018), it also benefited HCPs in terms of managing the potential risks that detainees were considered to present.

## Discussion

Healthcare in police custody is dissimilar to care in other institutions; nurses found that they needed to develop strategies for saying no and over time considered themselves to have become less empathic than their colleagues working in hospitals. This attitude was partly due to their characterisations of the people they were treating, for instance the expectation that detainees would be manipulative, aggressive and high maintenance in contrast to vulnerable hospital patients. The coldness expressed by HCPs was also a product of the different outcomes and objectives that they were expected to meet. Care in custody is not about fixing the patient (Rees 2020), but rather about managing risk and ensuring detainees are fit to be detained and interviewed. As a result, nurses expressed the need to maintain a distance from the detainees, which contrasted with other healthcare environments.

The framing of custody around criminal justice outcomes and the development of a distanced attitude towards detainees is not, however, evidence of a lack of care; care practices are coproduced alongside nurses’ criminal justice obligations (for instance those stated in PACE guidelines). Nurses have developed expertise in identifying when an alcoholic is sufficiently functional to be interviewed but not yet sober enough to enter withdrawal. Constructing this ‘Twilight Zone’ allows evidence collection from detainees at risk of alcohol withdrawal. Similarly, a polite and personable manner achieves compliance from the detainee. Other mundane expressions of care are also coproductions; for instance, collecting medication for a detainee allows the detained person to feel recognised and cared for, but at the same time reduces the risk of harm following release. HCPs have coproduced routines and activities that meet their care and evidence collection objectives, and as a result, rather than existing in a state of role-conflict, they can perform forensic work unproblematically (Mulla 2011).

Understanding care as an inherent part of criminal justice processing does not mean that it also needs to be wholly empathic and positive. Studies from the Sociology of Health and Illness have demonstrated how practices of care produce typification of patients, prioritising some for care over others. This was clearly evidenced amongst the HCPs, especially with respect to the treatment of drug and alcohol-dependent detainees. While alcoholics were constructed as highly vulnerable, drug addicts were seen as manipulative, high-maintenance and difficult to deal with. Nurses were advised of this characterisation in their training, and this carried through to their routine practices of care, resulting in different treatment trajectories and priorities based upon the relative risk of a death in custody.

Understanding healthcare within police custody as the result of coproduced practices is also consistent with contemporary understandings of the custody space (Skinns *et al.* 2016). Custody does not constitute one particular objective; rather, the messy, often contradictory goals that need to be negotiated by police and detention officer teams require healthcare professionals to be flexible and pragmatic. The anxiety around deaths in custody promotes a risk management approach to dealing with detainees, and HCPs have found ways to aid this by performing various forms of anticipatory work. For instance, they mobilise the space of the cell in situations where they expect the detainee to be violent or aggressive, and advise the Custody Sergeant about any potential healthcare risks that might also pose a risk to the custody (Rees 2020). In anticipating risks and collaborating with police and detention staff, HCPs’ coproduced practices assist the custody team in the accomplishment of all custody objectives.

Drawing attention to HCPs’ coproduced work challenges our current understanding of the care-custody paradox. Focusing on the coproduced nature of care work in custody leads to a reinterrogation of our previous understandings of forensic medical professionals. Based on this analysis, rather than HCPs being supplementary to the custody team, it appears that their anticipation and mundane care work are beneficial in terms of enabling police and detention staff to fulfil their objectives. I suggest, therefore, that rather than repeating the claim that there is a care-custody paradox, we acknowledge that forensic medical professionals are quite capable of coproducing their work to fit both their legal and medical responsibilities. As one HCP succinctly summed it up:

The job role doesn’t change, we are still here for the same purpose, we still have to work with PACE and NMC [Nursing and Midwifery Council professional codes of practice]. (Imogen D2)

## Conclusion

The care-custody paradox has been useful for highlighting some of the difficult decisions made by forensic medical professionals, but the extent to which they position healthcare and criminal justice as mutually exclusive has been significantly over-exaggerated. This examination of healthcare practice in police custody has highlighted that routine work is performed unproblematically, with local routines and procedures necessarily enabling staff to perform complex work without engaging in existential questions about their role or professional commitments (Rees 2015b). In this way, healthcare professionals collaborate with law enforcement colleagues in order to accomplish the objectives of criminal justice. Care is then coproduced between healthcare and legal objectives, and while this does result in HCPs’ attitudes changing – becoming ‘colder’ – there is still plenty of scope for professional, polite and considerate practices that enable detainees to feel treated as human beings (Skinns *et al.* 2020). This generates compliance from detainees and as such helps achieve the goals of the custody team.

It is important to reflect that this paper was an exploratory investigation of the role of care in police custody, and only HCPs were interviewed about their work; however, when comparing these findings with the work of Skinns and colleagues, there is substantial overlap between the HCPs’ narratives and detention/police officers’ discussions of dignified practices. Nevertheless, a larger study (including interviews and observation) is required to explore how all those within police custody (including detainees) interact, including the important role that materiality plays in these interactions, and to observe the division of labour between care and custody. It will then be possible to move beyond simply reconceptualising HCPs and provide the more thorough analyses necessary for the ambitious project of wholly reimagining care within police custody.

## Notes

1 Throughout this paper I will refer to HCPs and nurses interchangeably.

2 The PACE Codes of Practice set out suspects’ rights and entitlements during investigation, including whilst detained; for instance, access to free and independent legal advice and access to healthcare (Skinns 2011). As such, they also set out the responsibilities on police staff to ensure that these rights and entitlements are provided. PACE also places limits on the ways that evidence is gathered in a criminal investigation, and, particularly salient for this study, the temporal period of detention without charge, often referred to as the ‘PACE clock’ (Skinns 2011, p. 7).

3 It should be noted that the post-sexual assault forensic intervention of the victim-survivor is no longer performed in police custody suites in England and Wales, but instead in special units called Sexual Assault Referral Centres (SARCs) (Rees 2015a). The forensic examination of suspects, however, is still performed in custody suites.

4 The nomenclature of those detained in police custody is complex, as persons might be detained in connection with a criminal offence or for their own protection under S.136 of the Mental Health Act. In this paper I will use the terms most commonly employed by my respondents: ‘detainees’ or ‘detained persons’.

5 See Rees (2015b) for an example of such coproduction.

6 It is important to emphasise that police staff and detention officers were not involved in this study, again due to the exploratory nature of the project.

7 Ethics approval reference: 12617/2016.

8 Names provided are pseudonyms and are followed by an alphabetical representation of the constabulary they worked in and whether their contract was publicly (i.e. NHS) or privately provided. If the latter, a number represents the company.

9 The HCPs also discussed the interviews they performed with detainees accused of child sexual offences before release. The aim of these interviews was to risk-assess the likelihood that the detainee would harm themselves. There is not enough space here to discuss these assessments, but this again constitutes an example of the work of HCPs to mitigate post-release risk.

10 Amanda’s quotation implies a close and amiable working-relationship between HCPs and police colleagues, which impacts and influences criminal justice processes and decision-making. Nurses spoke at length of the need to develop strong working relationships with police colleagues and highlighted the difficulties that can result if they are not perceived by the police staff as being part of the custody team. I have explored these interpersonal aspects of interagency collaboration previously (Rees 2020).

11 HCPs’ professional construction of drug users here is similar to that of staff from drug and alcohol action teams interviewed by Bacon and Seddon (2020).
